# Role of vitamin B6 status on antioxidant defenses, glutathione, and related enzyme activities in mice with homocysteine-induced oxidative stress

**DOI:** 10.3402/fnr.v59.25702

**Published:** 2015-04-29

**Authors:** Cheng-Chin Hsu, Chien-Hsiang Cheng, Chin-Lin Hsu, Wan-Ju Lee, Shih-Chien Huang, Yi-Chia Huang

**Affiliations:** 1 School of Nutrition, Chung Shan Medical University, Taichung, Taiwan; 2 Department of Nutrition, Chung Shan Medical University Hospital, Taichung, Taiwan; 3 Critical Care and Respiratory Therapy, Taichung Veterans General Hospital, Taichung, Taiwan

**Keywords:** vitamin B6, glutathione, antioxidant enzyme activities, homocysteine-induced oxidative stress, mice

## Abstract

**Background:**

Vitamin B6 may directly or indirectly play a role in oxidative stress and the antioxidant defense system.

**Objective:**

The purpose of this study was to examine the associations of vitamin B6 status with cysteine, glutathione, and its related enzyme activities in mice with homocysteine-induced oxidative stress.

**Design:**

Four-week-old male BALB/c mice were weighed and divided into one of four dietary treatment groups fed either a normal diet (as a control group and a homocysteine group), a vitamin B6-deficient diet (as a B6-deficient group), or a B6-supplemented diet (a pyridoxine-HCl-free diet supplemented with 14 mg/kg of pyridoxine-HCl, as a B6 supplement group) for 28 days. Homocysteine thiolactone was then added to drinking water in three groups for 21 days to induce oxidative stress. At the end of the study, mice were sacrificed by decapitation and blood and liver samples were obtained.

**Results:**

Mice with vitamin B6-deficient diet had the highest homocysteine concentration in plasma and liver among groups. Significantly increased hepatic malondialdehyde levels were observed in the vitamin B6-deficient group. Among homocysteine-treated groups, mice with vitamin B6-deficient diet had the highest plasma glutathione concentration and relatively lower hepatic glutathione concentration. The glutathione peroxidase activities remained relatively stable in plasma and liver whether vitamin B6 was adequate, deficient, or supplemented.

**Conclusions:**

Mice with deficient vitamin B6 intakes had an aggravate effect under homocysteine-induced oxidative stress. The vitamin B6-deficient status seems to mediate the oxidative stress in connection with the redistribution of glutathione from liver to plasma, but not further affect glutathione-related enzyme activities in mice with homocysteine-induced oxidative stress.

Pyridoxal 5’-phosphate (PLP), the physiologically active coenzyme form of vitamin B6, is mainly involved in the metabolism of amino acids, nucleic acids, glycogen, porphyrin, and lipids. In addition, vitamin B6 may have a crucial role in antioxidant mechanism (1–6). Although the exact antioxidant mechanism of vitamin B6 has not been confirmed yet, vitamin B6 may directly react with the peroxy radicals and thereby scavenge radicals and inhibit lipid peroxidation (6–10). On the contrary, vitamin B6 may indirectly play an antioxidant role by serving as coenzyme in the glutathione antioxidant defense system. PLP serves as a coenzyme in the transsulfuration pathway of homocysteine to cysteine. Cysteine synthesized by this pathway is an important contributor to synthesis of reduced glutathione (GSH). The GSH-dependent antioxidant system, including glutathione peroxidase (GPx), glutathione reductase (GR), and glutathione *S*-transferase (GST), plays a fundamental role in cellular defense against reactive free radicals and other oxidant species (6, 11). A decrease in antioxidant enzyme activity may disrupt the balance between pro- and anti-oxidants, leading to higher oxidative stress and cellular damage. It would then be reasonable to hypothesize that deficient vitamin B6 status might either directly cause higher oxidative stress or might affect cysteine and GSH synthesis and, as a consequence, the entire GSH-dependent antioxidant defense system. A previous study showed increased malondialdehyde (MDA) level and GR activity, and decreased GSH synthesis, GPx, and GSH activities in liver tissue of vitamin B6-deficient rats (4). However, unchanged GSH concentrations and GR activities in the liver, kidney, brain, lung, spleen, and plasma, and increased GPx activities in the liver were observed in vitamin B6-deficient rats when compared to control rats (12). Other studies of animals (13, 14) and healthy humans (15) indicated that dietary vitamin B6 restriction did not affect liver/plasma cysteine concentrations but increased liver/plasma GSH concentrations. There seems to be an inconsistency regarding the relationship between level of GSH and its related enzyme activities and vitamin B6 status in animals.

Although increased oxidative stress has been observed in vitamin B6-deficient animal models (1, 2, 4), the antioxidant roles of vitamin B6 have not been fully studied yet. It is unclear whether deficient vitamin B6 status would mediate the increased oxidative stress in connection with deficient cysteine and GSH synthesis and decreased GSH-related enzyme activities. The purpose of this study was to examine the associations of vitamin B6 status with cysteine, GSH, and its related enzyme activities in mice with homocysteine-induced oxidative stress.

## Materials and methods

### Animals and diets

**Fig. 1:**
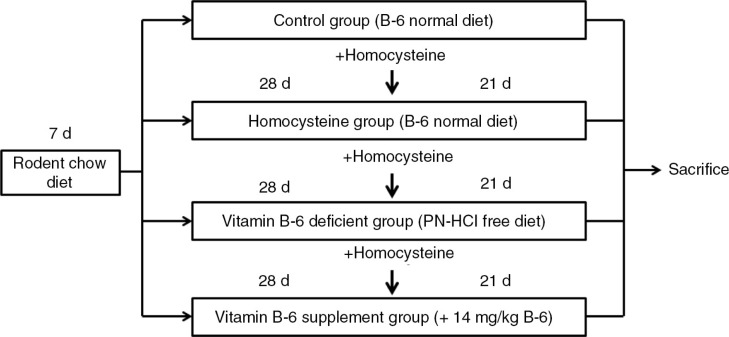
The study design.

Four-week-old male BALB/c mice were purchased from National Laboratory Animal Center (Taipei, Taiwan). Mice were housed in individual metal cages in an air-conditioned room at 23±2°C, 55–60% relative humidity, and a 12 h light/dark cycle, and were given a laboratory rodent chow diet for 7 days to allow for acclimatization. All procedures were approved by the Institutional Animal Care and Use Committee (IACUC) of Chung Shan Medical University (IACUC Approval No. 1092), Taichung, Taiwan. After 7 days of acclimatization, mice were weighed and evenly divided into one of four dietary treatment groups fed a normal diet (AIN-93-M, ICN Biomedicals, Inc., USA) (as a control group, *n*=9; and a homocysteine group, *n*=9), a vitamin B6-deficient diet (AIN-93-M without pyridoxine (PN)-HCl, ICN Biomedicals, Inc., USA) (as a vitamin B6-deficient group, *n*=9), or a vitamin B6-supplemented diet (a PN-HCl free diet supplemented with either 14 mg/kg) (as a vitamin B6 supplement group, *n*=9) for 28 days. The composition of normal and experimental diets is shown in Table 1. Hyperhomocysteinemia has been considered to be a potential oxidative stress indicator (16, 17) and has been used to induce oxidative stress in rats (18). Homocysteine thiolactone was then added to drinking water (1.8 g/L) for 21 days to induce oxidative stress (16, 17) in three groups (except for the control group). At the end of the study, mice were sacrificed by decapitation and blood and liver tissue samples were obtained. The study protocol is shown in Fig. 1.

**Table 1:** Composition of normal and experimental diets

Diet (ingredients)	%	Normal diet (g/kg dry matter)	Vitamin B6-deficient diet (g/kg dry matter)
Casein	14.00	140	140
Dextrinized cornstarch	15.50	155	155
Sucrose	10.00	100	100
Corn starch	46.60	466	466
Alphacel, non-nutritive bulk	5.00	50	50
Soybean oil	4.00	40	40
AIN-93M mineral mix^a^	3.50	35	35
l-Cystine	0.18	1.80	1.80
AIN-93-VX vitamin mix^b^	1.00	10	–
AIN-93-VX vitamin mix (without pyridoxine-HCl)	1.00	–	10
Choline bitartrate	0.25	2.50	2.50
*tert*-Butylhydroquinone	0.08×10^−2^	0.08×10^−1^	0.08×10^−1^

All compounds used in the study were of analytical grade.

a Supplied (g/kg diet), CaCO_3_, 357.00; KH_2_PO_4_, 250.00; K_3_C_6_H_5_O_7_·H_2_O, 28.00; NaCl, 74.00; K_2_SO_4_, 46.60; MgO, 24.00; Ferric citrate, 6.06; ZnCO_3_, 1.65; MnCO_3_, 0.63; CuCO_3_, 0.30; KIO_3_, 0.01; Na_2_O_3_Se, 10.25×10^−3^; (NH_4_)_6_Mo_7_O_24_·4H_2_O, 7.95×10^−3^; Na_2_O_3_Si·9H_2_O, 1.45; KCr(SO_4_)_2_·12H_2_O, 2.75×10^−1^; LiCl, 1.74×10^−2^; B(OH)_3_, 8.15×10^−2^; NaF, 6.35×10^−2^; NiCO_3_, 3.18×10^−2^; NH_4_VO_3_, 0.66×10^−2^; powdered sugar, 209.81.

b Supplied (g/kg diet), nicotinic acid, 3; D-calcium pantothenate, 1.6; pyridoxine-HCl, 0.7; thiamine HCl, 0.60; riboflavin, 0.60; folic acid, 0.20; D-biotin, 0.02; vitamin B-12 (0.1% triturated in mannitol), 2.50; α-tocopherol powder (250 U/g), 30.00; vitamin A palmitate (250,000 U/g), 1.60; vitamin D-3 (400,000 U/g), 0.25; phylloquinone, 0.75×10^−1^; powdered sucrose, 959.66.

### Biochemical measurements

During the experimental feeding, the animal weight and intakes were measured twice a week. Blood samples were withdrawn from inferior vena cava, transported on ice, and separated into plasma and red blood cells within 30 min by low speed centrifugation (3,000 rpm, 15 min, 4°C). Liver tissues were immediately homogenized in phosphate-buffered saline (PBS). The homogenized solution was then centrifuged (12,000 rpm, 4°C, 10 min). The supernatant were then carefully removed for analysis. All the samples were stored frozen (−20°C) until analysis.

Plasma and liver PLP concentrations were determined by high performance liquid chromatography (HPLC) as previously described (19). The inter- and intraassay variabilities of plasma and liver PLP were 5.1% (*n*=8) and 1.1% (*n*=5), respectively. Homocysteine and cysteine concentrations in plasma and liver were determined by HPLC using the method of Dudman (20). The interassay variabilities of homocysteine and cysteine were 6.2% (*n*=8) and 6.9% (*n*=8), respectively, and the intraassay variabilities of homocysteine and cysteine were 1.0 (*n*=5) and 0.9% (*n*=5), respectively. Vitamin B6 and homocysteine assays were carried out under yellow light to prevent photodestruction. Oxidative stress was estimated as the levels of plasma and liver MDA. Plasma and liver MDA was measured by thiobarbituric-acid-reactive substances (TBARs) according to a method previously described (21). The following reagents were used: PBS, 3% sodium dodecyl sulfate, 0.1 N hydrochloride, 10% phosphotungstic acid, 0.7% TBARs, and n-butanol. The excitation and emission wavelengths of fluorescence spectrophotometer (F-4500, Hitachi, Japan) were set at 515 and 555 nm, respectively. The GSH concentration in plasma and liver were measured using the method of Hissin and Hilf (22). Plasma or liver homogenates were diluted with PBS, mixed with 2% trichloroacetic acid, and then added to the reagent containing PBS-EDTA buffer, 5,5’-dithiobis-2-nitrobenzoic acid, nicotinamide adenine dinucleotide phosphate (NADPH), and GR. The enzyme-linked immunosorbent assay (ELISA) reader (PTL-3965, Jasco, Japan) was used to read the absorbance value at wavelength of 405 nm. The inter- and intraassay variabilities of plasma GSH were 3.5% (*n*=3) and 2.7% (*n*=5), respectively. The GSH-related enzyme (GPx and GR) activity levels in plasma and liver were measured using the method of Lawrence and Burk (23). For the analysis of GPx activity level, plasma or liver homogenates were diluted with PBS and NADPH, added to potassium phosphate buffer (PPB), GSH and GR, and then mixed with hydrogen peroxide. For the analysis of GR activity level, plasma or liver homogenates were mixed with PPB, NADPH, oxidized GSH, and hydrogen peroxide. The ELISA reader (PTL-3965, Jasco, Japan) was used to read the absorbance value at wavelength of 340 nm for GPx and GR activity levels. The interassay variabilities of plasma GPx and GR activities were 4.4% (*n*=3) and 6.8% (*n*=3), respectively, and the intraassay variabilities GPx and GR activities were 6.1% (*n*=5) and 6.2% (*n*=5), respectively. Protein content was determined using the method of Lowry et al. (24). Results were expressed in nmol/mg protein for GSH and nmol/min/mg protein for GPx and GR activity levels. All analyses were performed in duplicate.

### Statistical analyses

Data were analyzed using the SAS statistical software (version 9.3; Statistical Analysis System Institute Inc., Cary, NC, USA). A Kolmogorov–Smirnov test was performed to determine the normal distribution. Biochemical values are compared for significant differences using one way analysis of variance among groups. Because some data were skewed rather than normally distributed, differences among groups were then determined using the Kruskal–Wallis one way analysis of variance on ranks. Student–Newman–Keuls test was used for the post-hoc analysis. Correlations of PLP with cysteine, GSH, and antioxidant enzyme activities were determined using the Pearson correlation coefficient. Results were considered statistically significant at *p*<0.05. Values presented in the text are means±standard deviation (SD).

## Results

Table 2 shows the PLP, homocysteine, and cysteine concentrations in plasma and liver. Mice fed the vitamin B6-deficient diet had significantly decreased plasma PLP concentrations among groups. On the contrary, plasma PLP significantly increased when the vitamin B6-deficient diet was supplemented with 14 mg/kg of vitamin B6. Although control and homocysteine groups had adequate vitamin B6 intake, plasma and liver PLP concentration significantly decreased in the homocysteine group with homocysteine added to the drinking water. Homocysteine-treated mice had significantly higher homocysteine concentration in plasma and liver when compared with mice not given homocysteine. Among the homocysteine-treated groups, mice with the vitamin B6-deficient diet had the highest homocysteine concentrations in plasma and liver when compared with those of mice with normal diet or vitamin B6-supplemented diet. Plasma cysteine concentrations were significantly reduced in the vitamin B6-deficient group when compared with other groups, but there were no significant changes in cysteine concentrations in liver.

**Table 2:** Vitamin B6, homocysteine, and cysteine in plasma and liver

	Control (*n*=9)	Homocysteine (*n*=9)	B6 deficient (*n*=9)	B6 supplement (*n*=9)
PLP (nmol/L)				
Plasma	243.98±30.36^b^	224.51±15.83^c^	26.43±3.67^d^	276.58±16.22^a^
Liver	6.66±1.28^a^	4.90±0.90^b^	3.73±0.60^b^	4.94±1.33^b^
Homocysteine (µmol/L)				
Plasma	3.53±0.81^c^	15.43±1.93^b^	55.41±25.23^a^	21.27±4.74^b^
Liver	1.32±0.19^d^	2.14±0.21^b^	3.08±1.10^a^	1.79±0.15^c^
Cysteine (µmol/L)				
Plasma	117.73±21.34^a^	120.42±12.80^a^	95.80±10.37^b^	112.46±11.26^a^
Liver	37.39±11.36	32.01±5.03	28.19±3.81	27.54±5.79

PLP, pyridoxal 5’-phosphate. Values with different superscript letters are significantly different among groups; *p<*0.05.

Concentrations of the oxidative stress indicator, GSH, and its related enzyme activities are listed in Table 3. There were no significant changes in plasma MDA concentration among groups. Significantly increased hepatic MDA levels were only observed in the vitamin B6-deficient group. Among homocysteine-treated groups, mice with vitamin B6-deficient diet had the highest plasma GSH concentration and relatively lower hepatic GSH concentration. The GPx activities remained relatively stable in plasma and liver whether vitamin B6 was adequate, deficient, or supplemented.

**Table 3:** Oxidative stress indicator, glutathione, and antioxidant enzyme activities in plasma and liver

	Control (*n*=9)	Homocysteine (*n*=9)	B6 deficient (*n*=9)	B6 supplement (*n*=9)
MDA (µmol/L)				
Plasma	3.53±0.64	3.43±0.43	3.50±1.13	3.40±0.46
Liver	1.40±0.47^b^	1.37±0.46^b^	3.33±1.45^a^	1.18±0.55^b^
GSH (nmol/mg protein)				
Plasma	0.56±0.20^a^	0.32±0.09^c^	0.43±0.18^b^	0.26±0.03^c^
Liver	15.39±3.15^a^	11.50±0.79^b^	9.07±1.26^c^	9.72±2.12^c^
GPx (nmol/min/mg protein)				
Plasma	21.21±2.02	20.89±3.84	21.51±1.36	22.44±2.81
Liver	332.94±43.31	299.11±47.93	315.31±18.69	348.57±55.29
GR (nmol/min/mg protein)				
Plasma	3.27±0.73^a^	2.15±0.48^b^	2.63±0.55^b^	2.33±0.77^b^
Liver	77.06±6.02^b^	76.78±5.17^b^	84.51±3.64^a^	77.90±2.50^b^

MDA, malondialdehyde; GSH, reduced glutathione; GPx, glutathione peroxidase; GR, glutathione reductase.

Values with different superscript letters are significantly different among groups; *p<*0.05.

Plasma and hepatic PLP significantly negatively correlated with plasma (*r*=−0.74, *p*<0.001) and hepatic (*r*=−0.46, *p*<0.01) homocysteine concentrations, respectively. Hepatic PLP significantly negatively correlated with hepatic MDA (*r*=−0.35, *p*<0.01) levels. Plasma PLP positively correlated with plasma cysteine (*r*=0.41, *p*<0.05) concentration. Hepatic PLP positively correlated with hepatic cysteine (*r*=0.45, *p*<0.01) and hepatic GSH (*r*=0.48, *p*<0.01) concentration. However, plasma or hepatic PLP did not correlate with plasma or hepatic GPx and GR activities.

## Discussion

A vitamin B6-deficient diet has been reported to increase plasma lipid peroxidation (TBARS) levels (1, 2, 4). Supplementation of vitamin B6 to a folic-acid-deficient diet with excess methionine prevented the elevation of oxidative stress markers (i.e. serum TBARS and advanced oxidation protein products levels) in homocysteinemic rats (18). The results of the present study were in accordance with previous findings (1, 2, 18). Homocysteine-induced oxidative stress could be moderated by an adequate vitamin B6 diet or a B6-supplemented diet but was aggravated by a vitamin B6-deficient diet.

Vitamin B6 has been shown to prevent the oxygen radical generation and lipid peroxidation caused by H_2_O_2_ in U937 monocytes (10) and endothelial cells (25). Keles et al., (6) evaluated lipid peroxidation and free radical scavenging activities in kidney tissue of vitamin B6-deficient rats; the results showed that levels of total and non-enzymatic superoxide scavenger activity and antioxidant potential in kidney tissue of vitamin B6-deficient rats were significantly lower than those of the control rats. A study of human indicated that lower plasma PLP was associated with higher urinary 8-hydroxydeixyguanosine (an oxidative damage marker) concentration in older Puerto Rican adults (26), which also supports the association between lower vitamin B6 status and higher oxidative stress. In the present study, a significant relationship between vitamin B6-deficient status and higher lipid peroxidation was observed in mice livers. Although we could not demonstrate whether vitamin B6 had a free radical scavenging activity in this study, a possible direct antioxidant mechanism of vitamin B6 might be that vitamin B6 compounds have both the hydroxyl and amine group substitution on a pyridine ring which can react with the peroxy radicals and thereby scavenge radicals and inhibit lipid peroxidation (6–10).

In addition to the potential direct free radical scavenging capability of vitamin B6, vitamin B6 status might affect cysteine and GSH synthesis and be indirectly involved in the antioxidant defense system. We observed a significant reduced plasma cysteine concentration in mice with a vitamin B6-deficient diet; however, the same observation did not exist in liver. This finding may indicate that plasma cysteine concentration would be rapidly affected by vitamin B6-deficient intake, while hepatic cysteine concentration could be counterbalanced by entering from GSH pool or being kept out of the taurine pool (15, 27–30). However, animal and human studies have reported that cystathionine synthesis is more susceptible to dietary vitamin B6 restriction than cysteine concentration in the transsulfuration pathway (15, 31). Since we did not measure cystathionine concentrations in mice plasma and liver, the relationships among cystathionine, cysteine, and vitamin B6 status could not be discussed in the present study. In contrast with the changes of cysteine in plasma and liver, significantly increased plasma GSH and decreased hepatic GSH concentrations were observed in mice with a vitamin B6-deficient diet. However, previous studies of animals (13, 14) and healthy humans (15) indicated that a dietary vitamin B6 restriction increased hepatic glutathione concentrations. A possible explanation for the increased plasma GSH synthesis might be that vitamin B6 deficiency induces greater oxidative stress (1, 2, 4, 6), which may trigger the elevation of GSH transport from the liver to plasma (32, 33). It is worth noting that the ratio of plasma GSH (nmol/mg protein) to hepatic GSH (nmol/mg protein) was 4.74% in the vitamin B6-deficient group, compared to 2.78% in the control and 2.67% in the vitamin B6-supplemented group. The results of the present study seemed to support the hypothesis that the redistribution of GSH from liver to plasma could have occurred in the vitamin B6-deficient state.

Marginal vitamin B6 contents not only increased lipid peroxidation but also considerably stimulated the activity of GSH-related enzymes (2, 4, 6). In contrast to the previous findings (2, 4, 6), our results showed that GPx and GR activities were not correlated with the changes of vitamin B6 status. A previous study also indicated that the GR activities were not altered by vitamin B6 deficiency in rat tissues (12). In the antioxidant defense system, superoxide dismutase (SOD) is the first line of defense against oxygen free radicals, and it catalyzes the dismutation of the superoxide anion into hydrogen peroxide. Hepatic cytosol SOD activities of B6-deficient rats have been observed to be lower when compared to those of control rats regardless of exercise (5). Although we did not measure SOD activities, we assume that SOD would respond more quickly than GSH-related enzymes to the vitamin B6-deficient or supplemented status under homocysteine-induced oxidative stress. Further study is warranted to investigate the responses of SOD, GPx, and GR activities to the homocysteine-induced oxidative stress under vitamin B6-deficient and supplemented status.

The data herein indicate that mice with vitamin B6-deficient intakes had a aggravate effect while mice with adequate or supplemented vitamin B6 intake had a protective effect under homocysteine-induced oxidative stress. The vitamin B6-deficient status seems to mediate the oxidative stress in connection with the redistribution of GSH from liver to plasma, but could not further affect GSH-related enzyme activities in mice with homocysteine-induced oxidative stress.
